# Fabrication and Characterization of Ceftizoxime-Loaded Pectin Nanocarriers

**DOI:** 10.3390/nano10081452

**Published:** 2020-07-24

**Authors:** Pawan Kumar, Vinod Kumar, Ravinder Kumar, Catalin Iulian Pruncu

**Affiliations:** 1 Department of Materials Science and Nanotechnology, Deenbandhu Chhotu Ram University of Science and Technology, Murthal 131039, India; pawankamiya@yahoo.in; 2 Department of Bio and Nano Technology, Guru Jambheshwar University of Science and Technology, Hisar 125001, India; rs170090180003@gjust.org; 3 Department of Mechanical Engineering, Lovely Professional University, Phagwara 144411, India; rav.chauhan@yahoo.co.in; 4 Mechanical Engineering Department, University of Birmingham, Birmingham B15 2TT, UK; 5 Mechanical Engineering, Imperial College London, Exhibition Rd., London SW7 2AZ, UK

**Keywords:** Ceftizoxime, pectin, solvent displacement, nanocarriers, antibacterial activity

## Abstract

Ceftizoxime (C_13_H_12_N_5_NaO_5_S_2_) is a parenteral, third-generationcephalosporin antibiotic used to treat bacterial infections including ear, nose, and throat infections. In this work, pectin has been used as a nanocarrier for ceftizoxime due to its high biocompatibility and non-toxicity with tunable surface properties. Ceftizoxime-loaded pectin nanocarriers (CPN) were successfully synthesized by the solvent displacement method. Optimization of nanoformulation was done by response surface methodology using Design-Expert software. The optimized formulation examined various in-vitro characterizations such as particle size, morphology, and FTIR studies. TEM results revealed irregular shape nanoparticles within the range of 29–110 nm. The in-vitro drug release using the dialysis method was performed after 24 h where nanoformulation showed sustained drug release. Drug-loaded nanoparticles revealed good antimicrobial activity against *Bacillus cereus*, *Bacillus polymyxa*, *Enterobacter aerogenes*, and *Pseudomonas aeruginosa*.

## 1. Introduction

The oral drug administration route is very common and convenient for drug delivery [1]. However, it is not always the right path for some effective drug constituents, such as NSAIDs, peptides, or proteins which may poorly be absorbed and damage the mucosa in the stomach [2,3]. Many scientists tried new excipients for the development of effective drug carrier systems [4]. These drug carrier systems are capable of delivering the drug at the specific site of action and at the required time with the required amount [5]. Nanocarrier-based targeted drug delivery systems enabled the effective delivery of drug agents than conventional drug delivery systems such as solutions, lotions, creams, ointments, powders, suppositories, suspensions, injectable, pills, capsules and tablets [6]. Nanotechnology could play a fundamental role as nano-systems have shown their potential as the ideal drug delivery systems for poorly soluble, low absorption and unstable drugs [7]. Polymers such as chitosan, gelatin, cellulose, pectin are used to protect drugs from the physiological environment and prolong the release of drugs to improve its stability [8]. They are also mostly used as stabilizers, taste-making agents, and proactive agents [9]. The lack of toxicity and the low production costs of pectin make them a good choice for drug delivery purposes [10]. Pectin is a water-soluble, high molecular weight complex polysaccharide used in oral drug delivery systems [11] such as gastro-retentive systems, colon-specific delivery systems and mucoadhesive delivery systems [12]. Due to its biodegradable and biocompatible nature, this polymer used in pharmaceutical [13,14] and food industries [14,15]. Such features make pectin attractive for the preparation of a new and effective drug delivery system for cancer treatment, a combined multiple-cargo system consisting of the hydrophobic drugs [11]. The properties such as easy dissolution in a basic aqueous medium and gel formation in the acidic environment are more favorable for drug delivery [16]. Nanotechnology-based new drug delivery systems can offer significant advantages over the traditional delivery mechanisms in terms of high stability, high carrier capacity, and feasibility of incorporation of both hydrophilic and hydrophobic substances [5,6,7,8,9,10,11,12,13,14,15,16,17]. The use of an ideal drug delivery system is decided primarily based on the biophysical and biochemical properties of the targeted drugs being selected for the use in pharmaceutical formulations or biomedical applications [18]. The conventional drug delivery system possesses some issues including limited bioaccessibility and less diffusion capacity into the outer membrane which can be overcome by using an ideal nano-drug delivery system [19]. Ceftizoxime is a third-generation cephalosporin that is highly effective against both Gram-negative and Gram-positive bacteria [20]. It directly acts on the bacterial cell wall and weakens the cell wall formation process by hindering the peptidoglycan formation [21]. The therapeutic levels of ceftizoxime in body tissues produce many adverse effects on the body such as vestibular, renal, and auditory toxicity. Sometimes, it also causes hypersensitivity reactions due to a high dose of the drug [22]. The therapeutic efficacy of ceftizoxime can be increased by developing nanoparticle-based drug delivery systems with minimum side effects [23]. This may be achieved using fabricating drug-polymer conjugate (a natural anionic polymer that binds with cationic moiety) [24]. A convenient and fast solvent displacement method was used for the synthesis of monodispersed drug-loaded polymeric nanoparticles [25,26]. In this research work, we prepared ceftizoxime-loaded pectin nanocarriers (CPN) using solvent displacement method and investigated various properties. This allows to obtain promising results to be used in medical application in order to control better the drug delivery.

## 2. Materials and Methods

### 2.1. Chemicals and Strains

Ceftizoxime, pectin, and nutrient agar were bought from HIMEDIA laboratories (P) (Ltd., Maharashtra, India). Calcium chloride (CaCl_2_) pellets and polyvinyl alcohol were procured from S.D. Fine Chemical Ltd., di-Octyl sodium sulfosuccinate (DOSS) and mannitol were acquired from Central Drug House (P) Ltd., New Delhi, India; dichloromethane (DCM) was procured from SRL Ltd.

The microorganisms (*Bacillus cereus*, *Bacillus polymyxa*, *Enterobacter aerogenes*, and *Pseudomonas aeruginosa*) used for the assay of antibacterial activity of polymeric nanoparticles are enumerated in the lab. All four strains used as a reference, acquired from the National Collection of Dairy Cultures (NCDC), NDRI, Karnal, India. To enhance the growth of a microorganism, nutrient broth (medium) was used. For the media sterilization, the pressure chamber (autoclave) was used for 20 min at 121 °C. 2% agar was added whenever a solid medium was required.

### 2.2. Nanoformulation Synthesis

**Figure 1:**
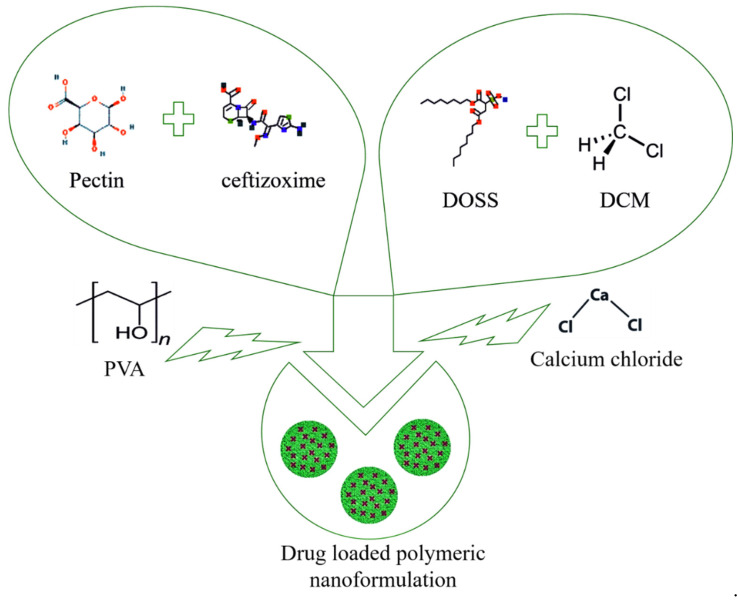
Schematic diagram of nanoformulation fabrication.

150 mg of ceftizoxime was mixed in 10 mL of 0.55% pectin solution. Di-octyl sodium sulfosuccinate (DOSS) solution was prepared by dissolving 5.50 g of DOSS in 100 mL of di-chloro methane (DCM). Add dropwise 30 mL solution of DOSS in the prepared solution under constant stirring for 10 min. 150 mL of 3% polyvinyl alcohol (PVA) was added and allowed for 10 min sonication. Then, the CaCl_2_ solution (20 g in 100 mL of DW) was dropwise, added in the solution under constant stirring for 12 h. After that, the solution was centrifuged at 12,000 rpm for 20 min. The filtrate was used to make pellets and dry in an electric oven at 60 °C for three hours. Figure 1 displayed the schematic diagram for ceftizoxime-loaded pectin nanocarriers.

### 2.3. Optimization of Nanoformulation

Pectin and di-octyl sodium sulfosuccinate were used as variables and their response was studied with Design-Expert v10. The response surface methodology (RSM) was performed for the given experiment. The concentrations of pectin and di-octyl sodium sulfosuccinate were considered as independent variables. The effects of these two variables were studied for particle size (nm) and the efficiency of encapsulation (%). These two factors were counted with 9 optimized runs as shown in Table 1. The designed experiment used runs to study the consequences of preparation variables on the particle size (that was taken as Y_1_) and % efficiency of encapsulation (that was made as Y_2_). The polynomial models included quadratic terms generated for the size of the particle and the ability of encapsulation. The 3D surface graphs were plotted using Design-Expert software.

### 2.4. Characterization

FTIR analysis was used for the confirmation of different functional groups present in the sample. The spectrum of nanoformulation was recorded using the frequency between 600–4000 cm^−1^ (Perkin Elmer Frontier FTIR, Boston, MA 02118, USA) with the combination of potassium bromide (KBr) pellets [27]. The size of particles and polydispersity index (PDI) of CPN were determined using particle size analyzer (Malvern Instrument, Enigma Business Park, Malvern WR14 1XZ, UK). The working principle of the system is based on quasi-elastic light scattering. Transmission electron microscopy (Hitachi H 7500, Chiyoda-ku, Tokyo, Japan) provides information onthe morphology of the sample exhibited by the generated good quality of 2D black and white images. UV–vis spectrophotometer (Shimadzu UV-2450, Milton Keynes, UK) was used to analyze the encapsulation efficiency of nanoformulation. Experiments were performed in triplicate and repeated three times with similar results.

### 2.5. Drug Loading and Efficiency of Encapsulation

The prepared nanoparticulate suspension was centrifuged at 10,000 rpm at −4 °C (4 K-15, Sigma, cooling centrifuge, Osterode, Germany). The obtained supernatant was analyzed by a UV-visible spectrophotometer that revealed absorbance at 298 nm for the unentrapped drug. Encapsulation efficiency (EE) was calculated by subtracting the amount of unentrapped drug in the supernatant from the total amount of the added drug. The amount of drug encapsulated in 0.1 g of nanoparticles is known as the loading of the drug. The standard curve equation was used for the determination of the encapsulation efficiency and loading of the drug [28].

(1) $$\mathrm{Encapsulation}\;\mathrm{efficiency}=\frac{\mathrm{Total}\;\mathrm{Drug}\;-\;\mathrm{Unentrapped}\;\mathrm{Drug}}{\mathrm{Total}\;\mathrm{Drug}}\times 100$$

### 2.6. In Vitro Drug Release

Pure drug and loaded drug nanoparticles were assessed through the dialysis technique [29]. Hot water was taken and dialysis bags were placed in that for 5 min before use. The bags were further used by rinsing the water. For calculation of dissolution efficacy, 30 mg CPN and 10 mg of ceftizoxime were placed in the dialysis membrane. The dialysis bags were immersed in a PBS solution and allowed for 24 h stirring. From the saturated solution, 4 mL of the sample were taken out every hour and the absorbance of the sample was observed using UV-vis spectrophotometry (at 298 nm).

### 2.7. Standard Calibration Curve

**Figure 2:**
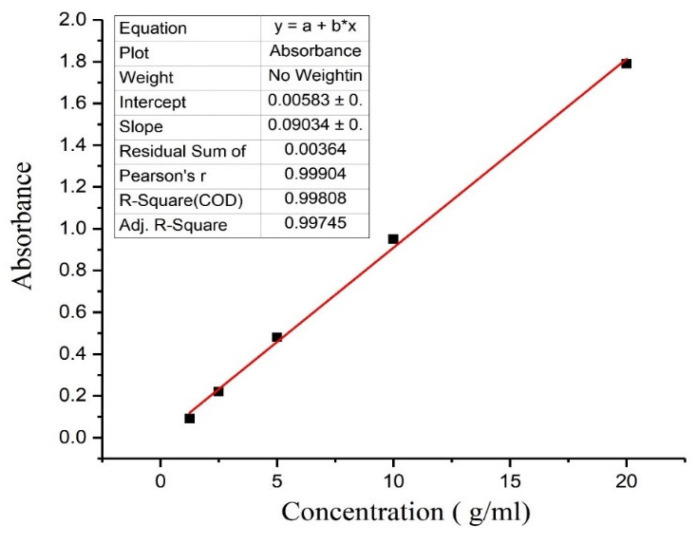
Standard curve of ceftizoxime.

A reliable, simple, and reproducible method was used for the estimation of drug content in an unknown sample by comparing the unknown to a set of standard samples of known concentration. To determine the concentration of ceftizoxime in the aqueous solution, concentrations of the drug against absorbance (λ_max_ 298 nm) were plotted to obtain a calibration curve. The method follows the Beer-Lambert law and measures the concentration of drugs between 1.25 to 20 μg/mL in distilled water. The achieved data of concentration and absorbance were linearly reverting; graph and line equations were calculated. The linearly reverted standard curve of ceftizoxime was plotted in Figure 2. The estimation procedure was found to be reasonably reproducible and fairly sensitive for the concentration range of 1.25–20 μg/mL. The related coefficient value in the standard graph was nearly 0.998. It shows that the drug obeys the Beer-Lambert law in the concentration between 1.25 to 20 μg/mL. The method is convenient, inexpensive, reproducible, and sensitive [30]. The experiment was repeated 3 times and the values are given in Table 2.

### 2.8. Optimization of Nanoformulation

As shown in Table 1, nine experimental runs were carried out for the optimization of the study. The designed experiment runs according to a standard protocol to find the changes in particle size (Y_1_) and % encapsulation efficiency (Y_2_). The polynomial models included quadratic equations generated for the size of particle and efficiency of encapsulation. These two factors were evaluated, each at three levels, and 9 experimental runs were carried out.

### 2.9. Cytotoxicity Study

In vitro cytotoxicity of the prepared nanoformulation was evaluated through the MTT assay [31,32]. A mouse fibroblast cell line (L929, ATCC) was used to ensure the biocompatibility of CPN by MTT (3-[4, 5-dimethylthiazole-2-yl]-2, 5 diphenyltetrazolium bromide) assay [33,34,35]. Briefly, cells were seeded at a density of 5 × 10^3^ cells/well in 96-well plates and cultured for 24 h in the incubator at 37 °C. The 5 µg/mL concentration of nanoformulation (CPN), ceftizoxime, and pectin were evaluated in comparison to control. Fibroblast cells were treated with 2 μg/mL concentration of aqueous formulation. The culture medium was replaced after 24 h with fresh media, and incubate for 1 h. After incubation for 1 h, the medium was discarded, the cells were washed twice with phosphate-buffered saline (PBS), and 50 mL of 5 mg/mL MTT solution in PBS were added to each well. The content of all wells was detached, and 150 μL of DMSO and 25 μL of Sorensen’s glycine buffer were added to each well to dissolve the substrate for 10 min. The absorptions were measured in triplicate at 570 nm using a microplate reader. Results were recorded as percentage absorbance relative to untreated control cells. The cytotoxicity assay results were calculated using Equation (2) [36].

(2) $$\%\;Cell\;Viability=\frac{\mathrm{the}\;\mathrm{absorbance}\;\mathrm{of}\;\mathrm{well}\;\mathrm{containing}\;\mathrm{sample}}{\mathrm{absorbance}\;\mathrm{for}\;\mathrm{untreated}\;\mathrm{control}\;\mathrm{cells}}\times 100$$

### 2.10. Antibacterial Activity

Antibacterial activity of CPN was evaluated against various bacterial strain viz., *Bacillus cereus*, *Bacillus polymyxa*, *Enterobacter aerogenes*, and *Pseudomonas aeruginosa*. For the antimicrobial study, the agar well diffusion method was used [37]. These bacteria were grown in lysogeny broth (LB) for 24 h, and 100 μL of the LB culture was used to spread over nutrient agar. This procedure uses paper disks (about 6 mm in diameter) impregnated with 20 μg ceftizoxime to test the susceptibility of microorganisms to ceftizoxime. The wells were filled with 50 μL suspension of CPN, paper disks (RD), pectin, and distilled water (DW), and incubated at 37 °C for 24 h and measured the zone of inhibition using a transparent ruler. The three replicates were used to evaluate the true error in the measured responses. These cultures were procured from the National Collection of Dairy Cultures (NCDC), NDRI, Karnal, Haryana, India. The cultures were maintained on nutrient agar for further use as per the condition is given in the MTCC protocol (Table 3).

## 3. Results and Discussion

### 3.1. Physiochemical Analysis

**Figure 3:**
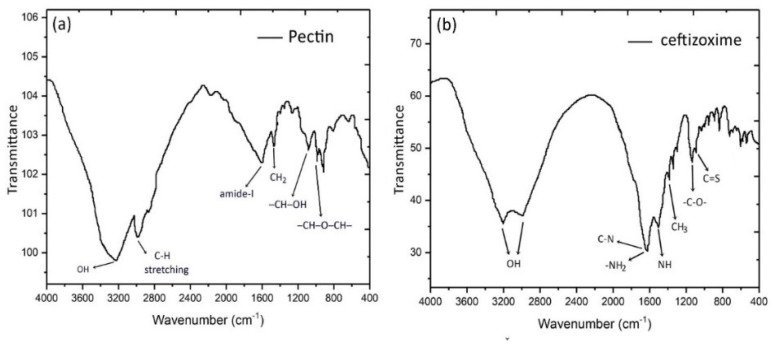
FTIR spectra of pectin (**a**) and ceftizoxime (**b**).

**Figure 4:**
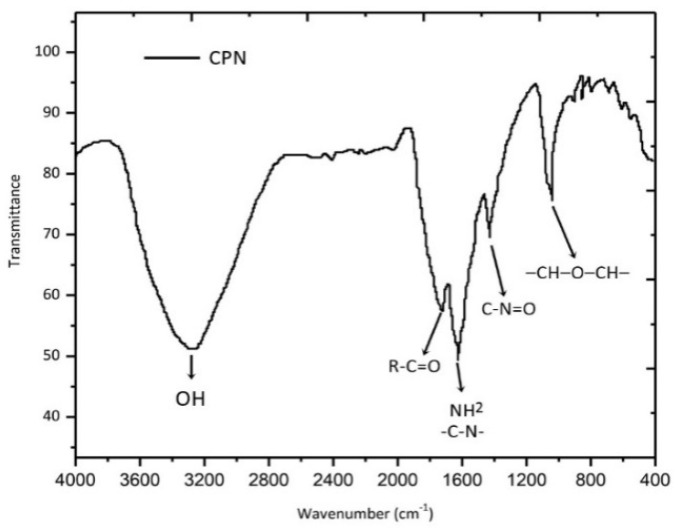
FTIR spectra of CPN.

FTIR spectrums of pectin, ceftizoxime, and CPN were displayed in Figure 3 and Figure 4. The spectra of pectin and ceftizoxime revealed the peaks at 3200–3000 cm^−1^ that confirmed the presence of an OH group. The chemical groups, along with the respective frequencies, are explained in Table 4. CPN revealed changes in phases that were recorded as the drug was entrapped in the pectin matrix.

**Figure 5:**
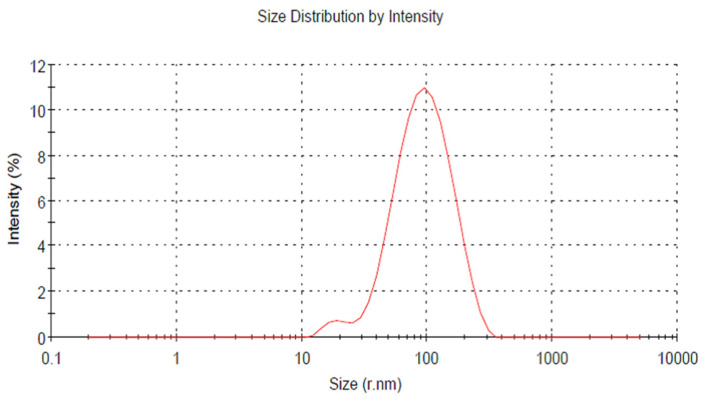
Particle size analysis of CPN (Ceftizoximeloade pectin nanocarriers).

The sample of CPN was lyophilized and dispersed in the double-distilled water with the help of the sonicator. The particle size determined by dynamic light scattering is as shown in Figure 5 and the average size of nanoparticles was found to be 99.5 ± 3.7 nm. The particle size analysis experiments were performed in triplicate.

### 3.2. Morphological Analysis

**Figure 6:**
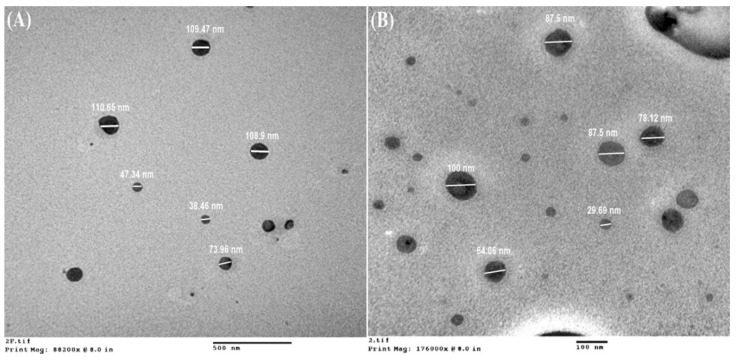
TEM images of CPN, subfigure (**A**) represents particles size at 500 nm scale and subfigure (**B**) represents particles size at 100 nm scale.

**Figure 7:**
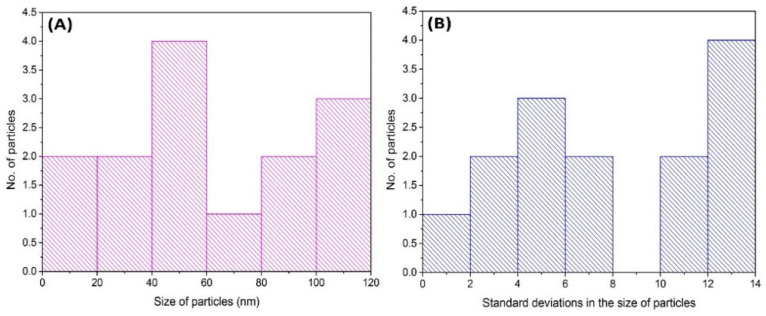
Particle size distribution histogram (**A**) and standard deviation (**B**) graph.

TEM images of polymeric nanoparticles were taken from the Hitachi H 7500, Chiyoda-ku, Tokyo, TEM system. Micrographs of TEM show the shape of CPN (Figure 6A,B), which is almost round in shape and nano in size. The results of TEM shows the size of the particle that is ranging from 70 nm to 100 nm. Figure 7 revealed the particle size distribution histogram and standard deviation graph. The histogram is a bar graph wherein the *x*-axis represents the CPN particle size and the *y*-axis represents the count.

### 3.3. Optimization of Nanoformulation

**Figure 8:**
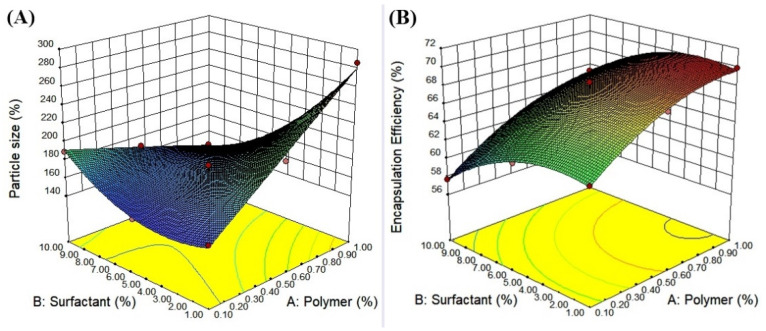
(**A**) Response surface graph showing the effects of pectin and di-octyl sodium sulfosuccinate (DOSS) concentration on the particle size and (**B**) the effectiveness of drug encapsulation in nanoparticles.

Table 1 revealed variables for initial experiments and speculative outcomes concerning the calculated variables on drug encapsulation efficiency and mean particle size. A mathematical relationship between factors and also parameters was shown by 3D surface graphs plots using Design-Expert software, as shown in Figure 8. The size of particles reduced when the concentration of the surfactant was boosted. All the PDI values for the 9 batches are within the acceptable limit. The encapsulation efficiency increased with increasing pectin concentrations and at higher values of di-octyl sodium sulfosuccinate. This is probably due to the formation of more nanoparticles by increasing the concentration of the surfactant. This also could be a result of the surfactant-induced reaction and interfacial tension between the two phases used. The positive effect on % EE may probably have occurred as a result of the ability of pectin to encapsulate large amounts of ceftizoxime due to an increase in the mass of pectin. Supplementary arithmetical considerations for all factors were scrutinized through the ANOVA test as depicted in Table 5. The results of the response surface methodology, best-fitted polynomial models, and ANOVA results are provided below:

(3) $$P.\;S.=+161.78-8.43\times A-2.68\times B+3.97\times \mathrm{AB}-38.47\times A^{2}-7.02\times B^{2}$$

(4) $$E.\;E.=+68.65+3.01\times A-2.00\times B+0.93\times \mathrm{AB}-2.52\times A^{2}-3.09\times B^{2}$$

The fitting outcomes showed that the optimized nanoparticles with high entrapment efficiency (69.44%) and lesser particle size (174.5 nm) were obtained with 1% pectin and 10%di-octyl sodium sulfosuccinate concentrations.

### 3.4. Drug Loading and Encapsulation Efficiency

The amount of drug loading in the pectin nanoparticles was determined by an ultraviolet-visible spectrophotometer. The unbounded drug was present in the supernatant, collected through centrifugation, and measured at 298 nm. The efficiency of encapsulation and loading of the drug was determined by the standard curve equation:

(5) y=11.04x−0.049

**Figure 9:**
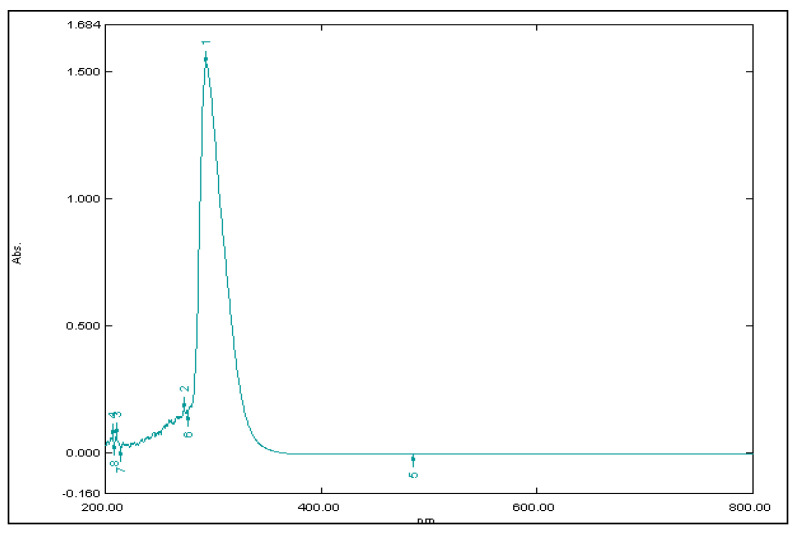
The UV-visible spectrum of CPN.

The peak of the supernatant of an optimized batch of CPN is shown in Figure 9. The total weight of nanoparticles was 3.69 gm, and the total amount of drug encapsulated was 97.06 mg. Drug loading was found to be 21% and encapsulation efficiency was counted as 69.4%.

### 3.5. In Vitro Drug Release

**Figure 10:**
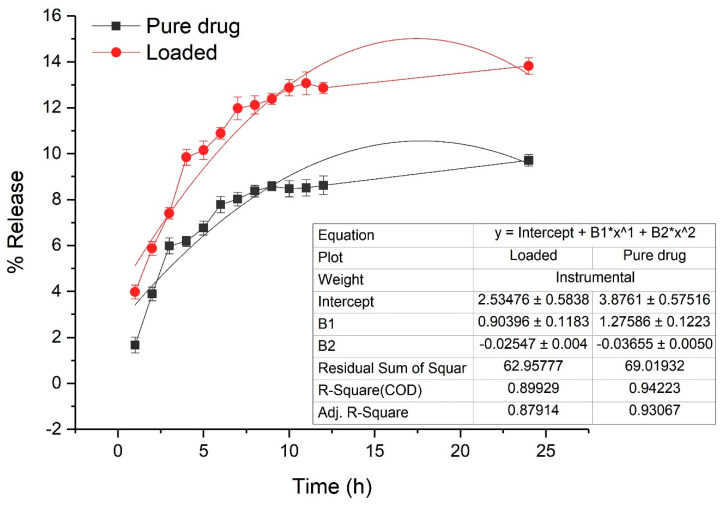
In vitro drug releases at different time intervals.

The drug release profile of the pure drug and the nanoparticulate system is provided in Table 4. Ceftizoxime escaped rapidly from the dialysis bag and showed the burst release from the nanoformulation that indicated the release of 9.71% after 24 h. The presence or entrapment of drug on the core of the polymer matrix makes formulation slow and release sustained. The comparative analysis of the drug release profile of ceftizoxime and CPN (Figure 10) indicated that the effect of ceftizoxime encapsulation in pectin provided a sustained release.

### 3.6. In Vitro Cytotoxicity

**Figure 11:**
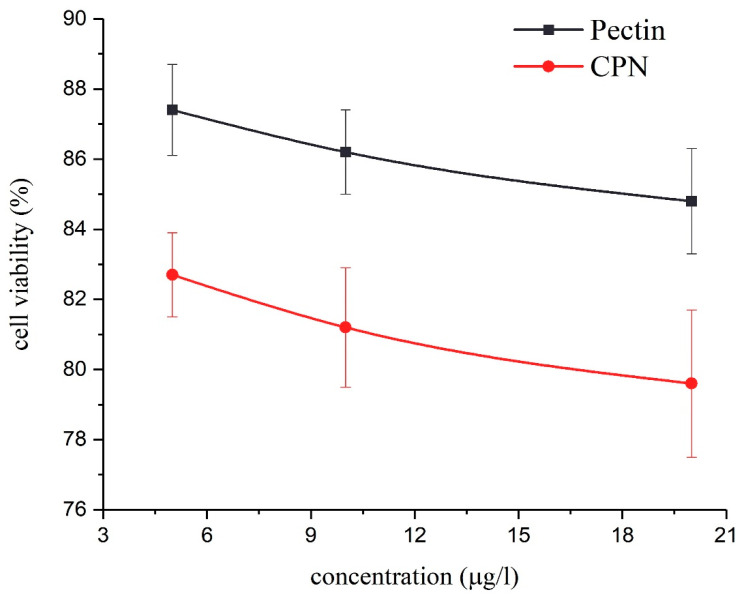
Comparative analysis of the percentage of cell viability against pectin and CPN.

The percentage viability of fibroblast cells incubated with nanoformulation (CPN) is shown in Figure 11. The CPN did not persuade any significant cytotoxic effect, even at the higher concentrations. As the concentration of CPN increases, the percentage of cell viability also decreased. For the comparative analysis, cytotoxicity of pectin (without drug) was also assessed against fibroblast cells using the MTT assay. Overall, both pectin and ceftizoxime-loaded pectin nanocarriers did not show significant toxicity against fibroblast cells.

### 3.7. Antibacterial Activity

**Figure 12:**
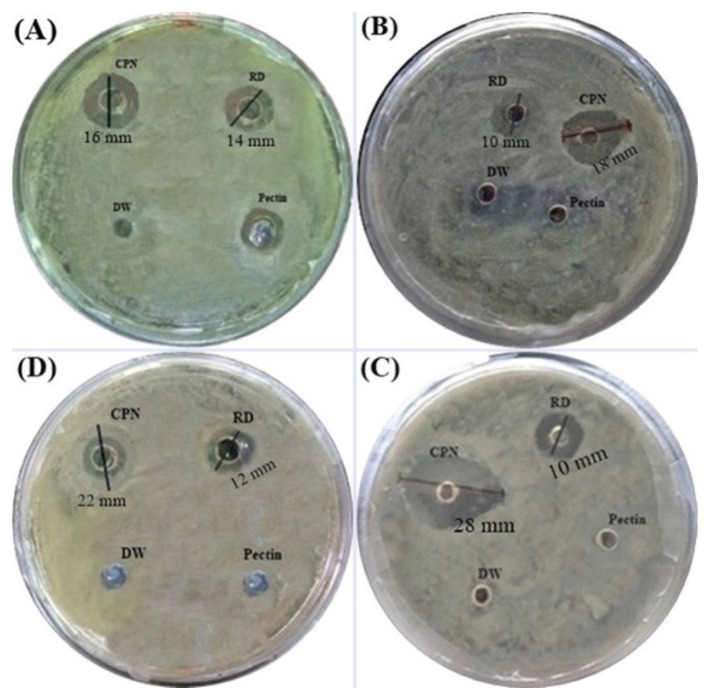
Zone of inhibition of CPN, and RD against *Bacillus cereus* (**A**), *Bacillus polymyxa* (**B**), *Enterobacter aerogenes* (**C**), and *Pseudomonas aeruginosa* (**D**).

The antibacterial activity of CPN and the reference drug (RD), ceftizoxime, against *Bacillus cereus*, *Bacillus polymyxa, Enterobacter aerogenes*, and *Pseudomonas aeruginosa*, are shown in Figure 12. The comparative zones of inhibition (ZoI) were analyzed, and the results are described in Table 6. CPN showed better antibacterial activity in comparison to the reference drug ceftizoxime and pectin. The highest activity was observed against *Enterobacter aerogenes*, as evident by the formation of an inhibition zone of 28 mm. The antibacterial analysis was performed in triplicate and then the inhibition zone was calculated.

## 4. Conclusions

In conclusion, ceftizoxime-loaded pectin nanoparticles (CPN) were successfully prepared using the solvent displacement method and were further optimized using a two-level factorial design. TEM analysis revealed 70–100 nm-sized round-shaped nanoparticles, whereas the presence of the drug in nanoparticles was confirmed with the aid of FTIR spectra. The drug loading in the pectin nanoparticles was found to be 21% and encapsulation efficiency was calculated as 69.4%. Antibacterial activity of ceftizoxime-loaded pectin nanoparticles showed a better zone of inhibition than the reference drug (ceftizoxime) because of the higher efficacy of the drug for a long time. CPN did not show any significant toxicity against fibroblast cell lines. In this way, ceftizoxime-loaded pectin nanocarriers can be used for their application in controlled drug delivery. However, further suitable animal model (in vivo) studies are still required to validate the results of in vitro evaluation and the use of CPNs for drug delivery applications.

## Figures and Tables

**Table 1:** Different experimental runs for optimization.

Sr. No.	Pectin (g) (X_1_)	Di–Octyl Sodium Sulfosuccinate (g) (X_2_)	Particle Size (nm) (Y_1_)	Encapsulation Efficiency (%) (Y_2_)
1	0.55	1.00	207.6	67.7
2	0.55	5.50	174.5	69.44
3	0.55	10.00	169.2	62.63
4	0.10	1.00	152.8	63.02
5	1.00	5.50	181.7	69.03
6	1.00	10.00	143.5	65.3
7	0.10	10.00	189.6	57.7
8	1.00	1.00	285.4	66.91
9	0.10	5.50	146.3	62.44

**Table 2:** Concentration vs. absorbance of ceftizoxime.

S. No.	Concentration (μg/mL)	Absorbance	Standard Deviation (SD)
1.	1.25	0.09	±0.002
2.	2.5	0.22	±0.035
3.	5	0.48	±0.09
4.	10	0.95	±0.14
5.	20	1.79	±0.33

**Table 3:** Bacterial strains.

Sr. No.	Name of Bacteria	Gram Staining Results	Accession Numbers
1.	*Bacillus cereus*	Gram positive	NCDC240
2.	*Bacillus polymyxa*	Gram-positive	NCDC 068
3.	*Enterobacter aerogenes*	Gram-negative	NCDC 106
4.	*Pseudomonas aeruginosa*	Gram-negative	NCDC 105

**Table 4:** FTIR spectra of pectin, ceftizoxime, and CPN [38,39,40].

Wavenumbers (cm^−1^)	Functional Groups
3300–3000	OH
2952	C−H
1715	R−C−O
1610	NH_2_, CH stretching
1598	amide-I
1490	NH stretching
1470	−CH_2_ scissoring
1420	C−N=O
1390	CH_3_
1180	−C−O−
1098	C=S
1080	−CH−OH bending vibration
1017	−CH−O−CH–

**Table 5:** Arithmetical consideration for optimization.

Model									Lack of Fit	
Response Factor	Prob. >*F*	*R* ^2^	*F* Value	Pred. *R*^2^	Adeq. Prec.	C.V.	Adjust. *R*^2^	Std. Dev.	Prob. >*F*	*F* Value
Y_1_	0.0045	0.9878	48.41	0.8817	21.322	4.30	0.9674	7.88	0.527	9.17
Y_2_	0.0029	0.9909	65.06	0.9266	23.517	0.96	0.9756	0.63	0.0180	20.30

**Table 6:** Zone of inhibition (ZoI) for RD vs. CPN with standard deviation.

Bacterial Strain	CPN	RD
*Bacillus cereus*	16 ± 1.5 mm	14 ± 0.3 mm
*Bacillus polymyxa*	18 ±1.8 mm	10 ± 0.2 mm
*Enterobacter aerogenes*	28 ± 2.3 mm	12 ± 0.2 mm
*Pseudomonas aeruginosa*	22 ± 1.9 mm	12 ± 0.1 mm
